# Parental knowledge, attitudes, and practices regarding laryngomalacia in Saudi Arabia: a nationwide cross-sectional study

**DOI:** 10.3389/fped.2026.1745657

**Published:** 2026-05-18

**Authors:** Hisham AlMutawa, Alya AlZabin, Aseel AlSaleh, Renad AlNofiey, Layan AlAmri, Fahad Z. AlOtaibi

**Affiliations:** 1 Department of Otolaryngology-Head and Neck Surgery, King Abdullah bin Abdulaziz University Hospital, Riyadh, Saudi Arabia; 2 College of Medicine, Princess Nourah Bint Abdulrahman University, Riyadh, Saudi Arabia; 3 Department of Otorhinolaryngology-Head and Neck Surgery, Imam Mohammad Ibn Saud Islamic University, Riyadh, Saudi Arabia

**Keywords:** attitude, children, knowledge, laryngomalacia, parents, practice, Saudi Arabia, stridor

## Abstract

**Background:**

Laryngomalacia (LM) is the most common cause of infant stridor, significantly impacting the quality of life for both patients and caregivers. Early recognition by parents is crucial to reducing morbidity and anxiety. However, parental knowledge, attitudes, and practices (KAP) regarding LM remain underexplored in Saudi Arabia.

**Methods:**

A nationwide cross-sectional study was conducted from December 2024 to July 2025 among parents residing in Saudi Arabia with at least one child aged ≥6 months. A validated, self-administered questionnaire assessed sociodemographics, knowledge, attitudes, and practices toward LM. Data were analyzed using descriptive statistics, chi-square, one-way ANOVA, and Spearman's correlation.

**Results:**

Of 1,537 participants, most were mothers (74.9%), aged 30–39 years (34.7%), and held a bachelor's degree (62.1%). Awareness was low, with 87.4% reporting they had never heard of LM. Knowledge gaps were evident, particularly in areas such as symptoms, diagnosis, and treatment. Positive attitudes were noted, with 76.9% agreeing that visiting a doctor for stridor is essential. In practice, 80.7% would seek medical care for stridor, while most rejected self-medication. Significant associations were found between KAP variables and sociodemographic factors, especially occupation, age, and residency. Knowledge correlated moderately with attitude (rho = 0.406, *p* < 0.001) and weakly with practice (rho = 0.196, *p* < 0.001).

**Conclusion:**

Parental awareness of LM in Saudi Arabia is limited despite positive healthcare-seeking attitudes. Tailored educational initiatives targeting identified knowledge gaps and misconceptions could enhance early diagnosis and management, ultimately improving outcomes for affected children.

## 1 Introduction

Laryngomalacia (LM) is the predominant cause of infant stridor, accounting for 54%–75% of cases (1). Common symptoms of LM include inspiratory stridor that exacerbates with eating, crying, supine lying, and agitation (2). LM was initially thought to be caused by immature cartilage; however, it is now believed to result from hypotonia triggered by abnormal sensorimotor integrative function, and this hypotonia allows prolapse of the supraglottic airway, leading to obstructive airway symptoms (3). Symptoms generally manifest shortly after birth, reach their peak between 6 and 8 months, and often resolve spontaneously within 12–24 months (4). LM may manifest independently but is commonly linked with other medical illnesses, such as gastroesophageal reflux disease, neurological disorders, congenital heart defects, congenital anomalies, and genetic disorders (5). The majority of LM cases are self-limiting or effectively managed with medical therapy; however, approximately 20% of newborns with LM experience severe illness necessitating surgical intervention (6). Chronic illnesses in childhood can significantly impact family well-being, leading to substantial economic, social, and psychological burdens. LM has a significant impact on the quality of life (QoL) for both patients and their caregivers. A prospective study conducted by Thottam et al. (2016) employed the Infant and Toddler Quality of Life Questionnaire (ITQOL-SF47) to evaluate the influence of disease severity on health perception and daily life in a cohort of 72 infants diagnosed with LM (7). The research indicated a significant association between heightened clinical seriousness, especially in moderate disease or prolapsing cuneiforms, and the presence of swallowing dysfunction alongside reduced QoL scores. Additionally, parents of affected children experienced considerable emotional distress, exhibiting significantly lower scores in areas of perceived physical health and the emotional impact on parents when compared to the general population. Moreover, Conway et al. (2020) performed a comprehensive survey of participants in an online LM support group to investigate caregiver experiences and anxiety levels (6). The study indicated that all caregivers experienced anxiety associated with the diagnosis, with 64% identifying as entirely anxious. Caregiver distress was significantly linked to delayed diagnosis, inadequate communication with healthcare providers, and the number of physicians consulted before visiting a pediatric otolaryngologist. Furthermore, children with ICU admissions, those requiring feeding tubes, or who underwent surgical interventions were more frequently perceived by caregivers as having poor health. The findings highlight the significance of early identification and comprehensive parental education regarding LM to alleviate caregiver anxiety and enhance outcomes via prompt management and support interventions.

A knowledge, attitude, and practice (KAP) survey is an effective tool for assessing knowledge levels in a specific health domain. KAP surveys, first developed in the 1950s for family planning and population research, have become widely recognized as an effective method for examining health-related behaviors and practices, as their design, administration, analysis, and interpretation are straightforward (8). The lack of literature addressing parental recognition of laryngeal malformations is particularly troubling, as early identification of stridor, feeding difficulties, and failure to thrive can expedite referral and management, thus reducing morbidity. Therefore, this study aimed to explore the KAP among parents in Saudi Arabia toward LM. The questionnaire items were developed based on commonly reported clinical features, diagnostic approaches, and management strategies for laryngomalacia described in pediatric otolaryngology literature and previous KAP survey methodology. This exploration aimed to assess the current level of awareness and prevalent attitudes and practices, thereby providing valuable insights for the development of targeted educational and preventive strategies.

## 2 Materials and methods

### 2.1 Ethics statement and informed consent

The study was conducted in accordance with the guidelines of the Declaration of Helsinki and received approval from the Institutional Review Board (IRB) at Imam Mohammad Ibn Saud Islamic University (IRB log number: 160-2021). All participants signed informed consent forms before the study began. The research goals were explained to everyone, and it was emphasized that they could withdraw at any time without facing any consequences.

### 2.2 Study design, setting, and participants

An online-based cross-sectional study with national coverage was conducted in Saudi Arabia. Study participants were recruited from the five major regions, and data collectors residing in the corresponding areas distributed the survey to ensure a representative sample. We used convenience sampling, distributing the survey through social media (X, WhatsApp, and Instagram) and parenting groups. The study was conducted from December 2024 to July 2025. The Inclusion criteria included (1) Parents aged 18 years and older who have at least one child aged ≥6 months. This criterion was applied to avoid inclusion of very young infants in whom respiratory symptoms may not yet have manifested or stabilized, (2) currently residing in Saudi Arabia, and (3) agreed to participate and complete the questionnaire. No gender restrictions were enforced, and there was no upper age limit specified for children. The exclusion criteria included (1) Parents with children less than 6 months of age, (2) individuals who did not consent to participate, and (3) participants who did not complete the questionnaire. The sample size was calculated based on a power of 80%, a margin of error of 5%, and a confidence level of 95%. The minimum required sample size was 650 participants, determined using G-power software. The total sample included 1,537 participants; additional responses were accepted to account for potential incomplete responses and to ensure robust statistical analysis across different subgroups.

### 2.3 Reliability of the survey

The internal consistency of the questionnaire used to assess parents' knowledge, attitudes, and practices toward LM was evaluated using Cronbach's Alpha. The questionnaire underwent a pilot test among a small sample of parents to assess clarity and comprehension. The overall Cronbach's Alpha coefficient for the 22-item instrument was 0.747, indicating an acceptable level of reliability. The questionnaire was developed after reviewing relevant literature on laryngomalacia and previously published KAP surveys. The initial items were reviewed by pediatric otolaryngology experts to ensure content relevance and clarity before distribution. The full questionnaire used in this study is available upon reasonable request from the corresponding author.

### 2.4 Statistical analysis and data management

Data were translated from Arabic into English and screened for missing values. Statistical analysis was conducted using SPSS version 27. Descriptive statistics, including frequencies, percentages, means, and standard deviations, were calculated. Inferential analyses included chi-square tests, independent t-tests, and one-way ANOVA to evaluate differences in knowledge, attitudes, and practices according to sociodemographic variables. *post-hoc* pairwise comparisons were not performed, as the analysis aimed to identify general associations rather than specific group differences. Spearman's correlation was used to assess relationships between knowledge, attitudes, and practices. Overall knowledge, attitude, and practice scores were calculated as the mean of the corresponding Likert-scale items within each domain.

## 3 Results

Table 1 presents the sociodemographic characteristics of the 1,537 participating parents. Most respondents were mothers (74.9%, *n* = 1,151), with fathers accounting for 25.1% (*n* = 386). Most participants were aged between 30 and 39 years (34.7%, *n* = 534), followed by those aged 40–49 years (30.3%, *n* = 466). Regarding educational level, a substantial proportion held a bachelor's degree (62.1%, *n* = 955), while 22.3% (*n* = 343) completed high school and 10.7% (*n* = 165) had postgraduate education. Participants were predominantly from the Central region (48.8%, *n* = 750), followed by the Western region (37.3%, *n* = 573). In terms of occupation, more than half were housewives (56.8%, *n* = 873), with 30.1% employed and smaller proportions being students (3.7%, *n* = 57), unemployed (3.9%, *n* = 60), or retired (5.5%, *n* = 85). The average number of children per respondent was 3 (SD = 2), with a range from 1 to 13. Among participants, 95.8% (*n* = 1,472) reported that their child had not been diagnosed with laryngomalacia, while 4.2% (*n* = 65) reported a diagnosis. Additionally, 87.4% (*n* = 1,343) had never heard of laryngomalacia. Regarding stridor, 1,229 parents (80%) stated that their child had never experienced it, whereas 308 (20%) reported a history of stridor.

**Table 1:** Participating parents’ sociodemographic characteristics, *N* = 1,537.

Item		*N*	%
Respondent	Mother	1,151	74.9
	Father	386	25.1
Age group	18–29	232	15.1
	30–39	534	34.7
	40–49	466	30.3
	50–59	252	16.4
	60–69	53	3.4
Level of education	Primary school	20	1.3
	Intermediate school	54	3.5
	High school	343	22.3
	Bachelor	955	62.1
	Postgraduate	165	10.7
Current region of residence	Central region	750	48.8
	Eastern region	120	7.8
	North region	20	1.3
	South region	74	4.8
	Western region	573	37.3
Occupation status	Housewife	873	56.8
	Employee	462	30.1
	Retired	85	5.5
	Unemployed	60	3.9
	Student	57	3.7
Number of children	Mean ± SD	3 ± 2	
	Median	3	
	Range (Min–Max)	12 (1–13)	

Table 2 displays the mean scores, standard deviations, and interpretations for each knowledge item regarding LM. The mean scores for most knowledge items were clustered around the “Neutral” range, indicating substantial uncertainty among respondents. Exceptions were the first three items, whether the child had been diagnosed with LM, whether the parent had heard about the condition, and whether the child had ever had stridor, which had very low mean scores and were interpreted as “Strongly Disagree.” The highest mean was for the statement “Diagnosis of LM can be made in the clinic” (Mean = 2.56, SD = 0.763), interpreted as “Agree.” Overall knowledge was rated as “Neutral” (Mean = 1.69, SD = 0.278), reflecting limited awareness in the population.

**Table 2:** Mean scores of parental knowledge items on laryngomalacia.

Knowledge items	Mean	Standard deviation	Interpretation
Has your child been diagnosed with laryngomalacia?	0.04	0.201	Strongly disagree
Have you ever heard about laryngomalacia?	0.13	0.332	Strongly disagree
Has your child ever had stridor?	0.20	0.400	Strongly disagree
Stridor means the child has adenoid hypertrophy.	2.19	0.704	Neutral
Stridor means the child has laryngomalacia.	2.15	0.583	Neutral
If my child has stridor, it means he has bronchial asthma.	1.98	0.725	Neutral
LM cause feeding difficulties.	2.28	0.721	Neutral
LM is more common in children less than two years of age.	2.21	0.638	Neutral
If LM is left untreated, it will never resolve.	1.95	0.698	Neutral
Diagnosis of LM requires the child to be sedated.	1.99	0.641	Neutral
Diagnosis of LM can be made in the clinic.	2.56	0.763	Agree
Treatment of LM is mainly surgical.	2.03	0.630	Neutral
Treatment of LM is mainly medical.	2.24	0.677	Neutral
Overall knowledge	1.69	0.278	Neutral

Table 3 presents the mean scores, standard deviations, and interpretations for parental attitudes toward LM. Parents generally demonstrated positive attitudes toward seeking medical care for children with stridor. The highest mean score was for “I would advise my relatives to visit the doctor if their children have stridor” (Mean = 3.18, SD = 0.808), followed closely by “Visiting the doctor is essential if your child has stridor” (Mean = 3.14, SD = 0.835), both interpreted as “Agree.” The statement “A child with LM will need medicine” was also rated as “Agree” (Mean = 2.54, SD = 0.799). Conversely, the belief that “LM may disappear by only observing the child” received a lower mean score (Mean = 2.10, SD = 0.773), interpreted as “Neutral.” Overall attitude was “Agree” (Mean = 2.74, SD = 0.549).

**Table 3:** Mean scores of parental attitudes toward laryngomalacia.

Attitude item	Mean	Standard deviation	Interpretation
Visiting the doctor is essential if your child has stridor	3.14	0.835	Agree
LM may disappear by only observing the child	2.10	0.773	Neutral
A child with LM will need medicine	2.54	0.799	Agree
I would you advise my relatives to visit the doctor if their children have stridor	3.18	0.808	Agree
Overall attitude	2.74	0.549	Agree

Table 4 outlines the mean scores, standard deviations, and interpretations for parental practices in managing symptoms or complications related to LM. Parental practices varied substantially. The strongest agreement was observed for “If my child is having stridor, I will seek medical advice” (Mean = 3.23, SD = 0.824, “Strongly Agree”), indicating a preference for professional care. Self-medication practices received the lowest scores, with “If my child is having stridor, I will self-medicate my child at home” (Mean = 1.22, SD = 0.953) and “I will give nasal drops” (Mean = 1.59, SD = 0.958), both interpreted as “Disagree.” The use of nebulization (Mean = 1.91, SD = 0.803) and seeking a local therapist for feeding difficulties (Mean = 2.33, SD = 1.143) were both rated as “Neutral.” Overall practice was also “Neutral” (Mean = 2.06, SD = 0.529).

**Table 4:** Mean scores of parental practices toward laryngomalacia.

Practice item	Mean	Standard deviation	Interpretation
If my child is having stridor, I would prefer him to receive nebulization.	1.91	0.803	Neutral
If my child is having stridor, I will seek medical advice.	3.23	0.824	Strongly agree
If my child is having stridor, I will self-medicate my child at home.	1.22	0.953	Disagree
If my child is having stridor, I will give nasal drops.	1.59	0.958	Disagree
If my child has feeding difficulties, I would seek professional help to improve feeding.	2.33	1.143	Neutral
Overall practice	2.06	0.529	Neutral

Table 5 demonstrates the relationship between selected sociodemographic characteristics and knowledge variables of parents towards LM. The One-Way ANOVA analysis indicated that knowledge items related to LM, specifically knowledge of the disorder and its differentiation from other causes of stridor (e.g., asthma, adenoids), were significantly associated with the age and occupation of the parents. Besides, the understanding of diagnosis and the interpretation of symptoms were two concepts that residency influenced. In addition, the study results also suggest that parents' information regarding the normal age of onset and available treatments (surgical vs. medical) varied by residency and occupation.

**Table 5:** The relationship between sociodemographic characteristics and knowledge statements of parents towards LM using One Way ANOVA.

Knowledge statements	Age	Level of education	Current region of residence	Occupation status
Has your child been diagnosed with laryngomalacia?	0.165	0.166	0.444	0.911
Have you ever heard about laryngomalacia?	0.003*	0.384	0.040*	0.070
Has your child ever had stridor?	0.155	0.993	0.110	0.177
Stridor means the child has adenoid hypertrophy.	0.559	0.314	0.004*	0.562
Stridor means the child has laryngomalacia.	0.644	0.167	0.062	0.012*
If my child has stridor, it means he has bronchial asthma.	0.015*	0.522	0.539	0.002*
LM cause feeding difficulties.	0.184	0.552	0.057	0.001*
LM is more common in children less than two years of age.	0.701	0.806	0.004*	0.005*
If LM is left untreated, it will never resolve.	0.029	0.275	0.210	0.063
Diagnosis of LM requires the child to be sedated.	0.627	0.394	0.004*	0.610
Diagnosis of LM can be made in the clinic.	0.318	0.073	0.029*	0.014*
Treatment of LM is mainly surgical.	0.047*	0.385	0.232	0.197

* Statistically significant *p*-value less than 0.05.

Values represent *p*-values obtained from one-way ANOVA tests.

Table 6 presents the relationship between selected sociodemographic characteristics and attitude variables of parents towards LM. The One-Way ANOVA test results showed that parental attitude statements about LM were influenced by different sociodemographic characteristics, primarily occupation status, level of education, age, and current region of residence (*p* < 0.05). Among these, the idea that a doctor visit is crucial for a child with stridor varied significantly by both residency and occupation. The opinion that LM might improve just by watching the patient varied depending on the parents' work status. Moreover, the view of the necessity of drugs was strongly linked to all four demographic variables. The readiness to recommend a relative to a doctor in case of stridor also differed significantly by age and occupational status.

**Table 6:** The relationship between sociodemographic characteristics and attitude statements of parents towards LM using One Way ANOVA.

Attitude statements	Age	Level of education	Current region of residence	Occupation status
Visiting the doctor is essential if your child has stridor.	0.184	0.734	0.013*	0.000*
LM may disappear by only observing the child	0.577	0.410	0.298	0.016*
A child with LM will need medicine	0.002*	0.018*	0.020*	0.037*
I would you advise my relatives to visit the doctor if their children have stridor	0.040*	0.482	0.396	0.000*

* Statistically significant *p*-value less than 0.05.

Values represent *p*-values obtained from one-way ANOVA tests.

Table 7 presents the relationship between selected sociodemographic characteristics and parental practice variables regarding LM. The One-Way ANOVA analysis revealed that parental practices regarding LM were statistically associated with various socio-demographic variables. The most significant variable was occupation, which yielded a consistent and significant result across all practice items (*p* < 0.001). The choice of taking a child with stridor to the doctor was a medical decision that differed significantly by age and occupation. Nebulization and self-medication, respectively, were significantly affected only by the changes in the occupation variable. The methods of nasal drop administration were visibly linked with age, residency, and occupation. Moreover, the choice of visiting a local therapist for feeding difficulties significantly differed by age, education level, residency, and occupation.

**Table 7:** The relationship between sociodemographic characteristics and practice statements of parents towards LM using One Way ANOVA.

Practice statements	Age	Level of education	Current region of residence	Occupation status
If my child is having stridor, I would prefer him to receive nebulization.	0.184	0.893	0.268	0.000*
If my child is having stridor, I will seek medical advice.	0.034*	0.688	0.068	0.000*
If my child is having stridor, I will self-medicate my child at home.	0.105	0.891	0.368	0.000*
If my child is having stridor, I will give nasal drops.	0.010*	0.729	0.014*	0.000*
If my child has feeding difficulties, I would seek professional help to improve feeding.	0.003*	0.001*	0.008*	0.000*

* Statistically significant *p*-value less than 0.05.

Values represent *p*-values obtained from one-way ANOVA tests.

Table 8 displays the correlation between parents' knowledge, attitude, and practice towards LM using Spearman's rho. Spearman's correlation analysis showed that there is a moderate positive correlation between parents' knowledge and their attitude towards LM (Spearman's rho = 0.406, *p* < 0.001), which means that more knowledge is linked to more positive attitudes. There was also a weaker but statistically significant positive correlation between knowledge and practice (Spearman's rho = 0.196, *p* < 0.001), indicating that increased knowledge slightly impacts parents' caregiving behavior.

**Table 8:** Correlation between knowledge, attitude and practice of parents towards LM using spearman's rho.

Variable	Correlation coefficient (Spearman's rho)	p-value
Attitude	Correlation Coefficient	0.406*
	Sig. (2-tailed)	<0.001
Practice	Correlation Coefficient	0.196*
	Sig. (2-tailed)	<0.001

* Correlation is significant at the 0.01 level (2-tailed).

## 4 Discussion

LM is the leading cause of infant stridor and significantly affects child QoL and caregiver well-being. Most research focuses on clinical features and treatment, with little emphasis on parents’ awareness, attitudes, and caregiving, especially in Saudi Arabia. This gap is critical since parents are often the first to notice early signs like stridor or feeding problems, influencing how quickly a child gets medical help. Addressing this issue will provide valuable insights into the community, informing public health strategies, education, and clinical communication. This can lead to earlier diagnosis, less caregiver anxiety, and better child outcomes. We found a significant lack of awareness, with 87.4% of parents reporting that they had never heard of LM. However, these were not the only data sources indicating that parents were generally receptive to seeking healthcare, as over three-fourths of parents agreed that visiting a doctor for stridor is necessary, and 80% of them stated they would seek medical advice if their child developed stridor. Variations in sociodemographic factors such as age, occupation, and region of residence were positive indicators of knowledge and practices, highlighting the need for tailored interventions. Although housewives constituted the largest proportion of participants, occupation remained a significant factor associated with KAP outcomes. This may reflect differences in exposure to health information, social networks, educational opportunities, and access to healthcare resources among occupational groups. Future studies should further explore how employment status influences parental health literacy and healthcare-seeking behaviors. Knowledge was moderately related to attitude and weakly to practice, suggesting that improving parental knowledge could boost positive attitudes and behaviors. Notably, parents demonstrated low knowledge but positive attitudes towards healthcare, indicating that they may support campaigns and education for early diagnosis and reducing caregiver burden, despite their limited understanding.

Our nationwide survey found that only 12.6% of Saudi parents had ever heard of LM and that most respondents were unable to recognize its clinical features or management options. This limited awareness mirrors patterns seen in studies of other pediatric airway disorders. For example, a cross-sectional survey in Jeddah assessing knowledge of childhood obstructive sleep apnea (OSA) among 146 parents reported a mean knowledge score of 15.38 ± 6 indicating limited parental knowledge and found that only 20% of parents demonstrated good knowledge; 37.4% of respondents had never heard of OSA (9). Similarly, extensive surveys on foreign-body aspiration (FBA) noted that most Saudi parents have poor knowledge of choking risks and first-aid techniques. In a study from Makkah, 65.4% of 1,087 parents had poor knowledge, and 78.6% had inadequate preventive practices (10). Women and parents with a bachelor's degree were more likely to have good knowledge. Still, misconceptions persisted, for instance, many believed that x-rays detect all foreign bodies or that peanuts are safe for toddlers. Outside Saudi Arabia, only 15.6% of Croatian parents could list the significant symptoms of FBA, and a mere 2% provided an accurate first-aid response when asked (11). These studies collectively suggest that parental knowledge gaps are widespread across different respiratory conditions, reinforcing our observation that targeted educational initiatives are necessary.

Despite limited knowledge, parents held positive attitudes toward seeking medical care; more than 76% agreed that visiting a doctor for stridor is essential, and most rejected self-medication. Similar optimism has been reported elsewhere. In the Jeddah OSA survey, most parents believed that consulting a doctor and awareness campaigns would improve knowledge (9). However, this optimism rarely translates into appropriate practices. In Al-Qassim, 61.3% of parents scored poorly on knowledge and 55.3% on practices regarding FBA; many parents admitted they would attempt to remove a foreign body themselves (12). Even when parents perceive themselves as knowledgeable, their ability to act correctly is limited. A Croatian study found only a moderate correlation between perceived knowledge and actual performance, which was assessed through participants' ability to correctly identify symptoms and appropriate first-aid responses (11). Our findings, therefore, extend previous work by demonstrating that relatively cautious attitudes, despite poor knowledge about LM, may still result in inappropriate management decisions.

In our survey, parents who reported prior awareness of LM most commonly indicated social media and internet platforms as their primary sources of information. This reliance on online resources is concerning because the quality of content varies substantially. A recent assessment of 95 YouTube videos on LM found that caregivers produced nearly one-third of the videos, with many aiming to demonstrate symptoms. However, only 25.3% of the videos provided a complete overview of the condition (13). Hospital-produced videos achieved higher DISCERN scores, yet were less frequently viewed; 58.9% of videos satisfied only one of five reliability criteria. Moreover, a few videos cited scientific references. These findings suggest that parents may encounter misleading or incomplete information when searching online, which may contribute to knowledge gaps and anxiety. Healthcare providers should therefore guide families toward vetted educational materials and consider producing their own content to improve online quality.

Our study highlights parental knowledge deficits, but the literature indicates that healthcare professionals also require guidance. A national survey of 233 Spanish pediatricians found that their general knowledge of LM was high (≥89%) but declined to 57% when more complex management questions were posed (14). The majority (54.1%) favored expectant management in suspected LM cases, and 67% were aware of additional tests needed for severe cases. These data underscore the need for standardized guidelines and continuing medical education, particularly regarding severe LM. Improving clinicians' understanding will enable them to counsel parents more effectively and avoid conflicting advice.

Epidemiological data support our observation that parents in Saudi Arabia rarely encounter LM in daily life. A cross-sectional study from Nepal evaluating 430 infants with stridor found that LM accounted for 66% of cases; 67.6% were classified as mild, and the mixed type (combined anatomical features involving both epiglottic collapse and arytenoid prolapse) was the most common (7). A retrospective review of 52 Saudi children similarly reported that most cases were mild or moderate (42.3% and 53.8%), with 69.2% being full-term and 65.4% presenting within three months of life (15). Synchronous airway lesions were present in about one-fifth of patients, and only 15% required surgery. Together, these studies imply that although LM is the leading cause of infantile stridor, severe disease is relatively uncommon, a factor that may contribute to low parental awareness. However, even mild cases can create parental anxiety due to the characteristic inspiratory noise and feeding difficulties.

Our study did not assess disease severity, but understanding clinical–physiological correlations helps explain parental anxiety. A large retrospective cohort of 157 infants with LM found that severe cases presented at a younger age and were associated with higher apnea-hypopnea indices and elevated end-tidal carbon dioxide levels on polysomnography (16). Notably, the clinical severity did not correlate with the laryngoscopic type, suggesting that bedside assessment alone may underestimate physiologic impairment. These findings support recommendations to use objective measures in moderate-to-severe cases to guide management and counselling.

Beyond the immediate clinical features, LM affects the well-being of families. A prospective QoL assessment using the LM QoL survey showed that 26 families reported significant burden; mean QoL scores improved after treatment (from 2.57 to 1.67 in medically managed infants and from 3.59 to 2.22 after supraglottoplasty), with surgical patients starting at higher symptom scores but achieving similar QoL after follow-up (17). Complementary research into caregiver psychology reported that burnout levels decline as children age but increase with greater LM severity and comorbidities; caregivers with depression or anxiety exhibit higher burnout (18). These studies emphasize that the psychosocial impact of LM is substantial, even when physical symptoms are mild, and highlight the importance of providing emotional support alongside medical care. The findings from our survey and the broader literature converge on a common theme that early recognition and appropriate management of LM require collaboration between informed parents and knowledgeable clinicians.

This study presents several limitations that must be acknowledged when interpreting the results. The application of a convenience sampling method and online distribution through social media may have resulted in selection bias, potentially overrepresenting participants with internet access and elevated health awareness. The cross-sectional design captures parental knowledge, attitudes, and practices at a single point in time, which limits the assessment of changes over time or the establishment of causality between variables. Third, self-reported responses are prone to recall bias and social desirability bias. In particular, parents of older children may have reduced accuracy in recalling early-life respiratory symptoms compared with parents of younger children. Fourth, although the questionnaire underwent validation and cultural adaptation, some nuances of LM-related knowledge may not have been completely captured. Moreover, although the present study primarily used univariate statistical tests to explore associations between sociodemographic variables and KAP outcomes, future research could benefit from multivariable regression models to account for potential overlap between factors such as age, education, occupation, and region of residence. Finally, the study population was predominantly comprised of mothers and residents from the Central and Western regions, which may have limited the generalizability of the findings to fathers or less-represented geographic areas. Additionally, there may be overlap between respondent type (mother vs. father) and occupation status (e.g., housewife), as the majority of housewives were mothers. Therefore, the observed associations with occupation may partially reflect the influence of being the primary caregiver rather than occupation alone.

The importance of this research lies in its being the first nationwide study to assess the knowledge, attitudes, and practices of parents regarding LM in Saudi Arabia. By demonstrating significant knowledge deficits in addition to positive attitudes toward healthcare, this suggests a clear direction for culturally targeted educational interventions, which are likely to empower parents, support early diagnosis, and ultimately decrease caregiver distress and child morbidity. More studies are needed to assess the effectiveness of the interventions, methods for reaching those who are underrepresented, as well as longitudinal studies to determine whether the increase in parental knowledge impacts clinical outcomes across time.

## 5 Conclusion

In conclusion, this study highlights substantial knowledge deficits among parents in Saudi Arabia regarding LM, despite generally favorable attitudes and appropriate healthcare-seeking behaviors. Sociodemographic factors, particularly occupation, age, and residency, influence parental understanding and responses. Implementing targeted, culturally relevant educational programs, particularly through healthcare providers and community platforms, may bridge these gaps, promote earlier recognition of LM, and reduce caregiver distress. Future research should evaluate the effectiveness of such interventions in improving parental knowledge and clinical outcomes.

## Data Availability

The original contributions presented in the study are included in the article/Supplementary Material, further inquiries can be directed to the corresponding author/s.
